# Investigation of association between serum C‐reactive protein concentrations and proteinuria in dogs

**DOI:** 10.1111/jsap.70040

**Published:** 2025-10-29

**Authors:** E. Ruane, M. M. A. Rodgers, C. H. Z. Hare, K. E. McCallum, T. L. Williams

**Affiliations:** ^1^ Department of Veterinary Medicine University of Cambridge Cambridge UK

## Abstract

**Objectives:**

Identify if serum C‐reactive protein concentrations and specific diseases are associated with proteinuria (defined as urine protein:creatinine ratio >0.2) in dogs without known pathological pre‐renal, renal or post‐renal causes.

**Materials and Methods:**

Hypothesis generating retrospective study. Dogs with contemporaneous urine protein:creatinine ratio and serum C‐reactive protein concentrations and without known causes of pathological pre‐renal, renal or post‐renal causes of proteinuria were included. Continuous and categorical variables were compared between groups using non‐parametric statistics, and multivariable logistic regression analyses evaluated associations between specific diseases or selected clinicopathological variables (including serum C‐reactive protein concentrations) and proteinuria.

**Results:**

Seventy‐one overtly proteinuric (urine protein:creatinine ratio >0.5), 74 borderline proteinuric (urine protein:creatinine ratio 0.21 to 0.5) and 234 non‐proteinuric dogs (urine protein:creatinine ratio ≤.2) were included. Proteinuria (urine protein:creatinine ratio >0.2) was less prevalent in dogs diagnosed with chronic enteropathy (11% [4/35] vs. 41% [141/344]; *P* < .001) compared to the rest of the population. Proteinuria was more prevalent in dogs with chronic hepatitis (71% [10/14] vs. 37% [135/365]) and tended to be more prevalent in dogs with pancreatitis (57% [12/21] vs. 37% [133/358]; *P* = .103) compared to the rest of the population. On multivariable analysis, serum C‐reactive protein concentration was independently associated with increased odds of proteinuria (OR = 1.031 [95% CI: 1.012 to 1.051]; *P* = .001) and a diagnosis of chronic enteropathy was independently associated with decreased odds of proteinuria (OR = 0.21 [95% CI: 0.064 to 0.681]; *P* = .009).

**Clinical Significance:**

Systemic inflammation might be associated with proteinuria in dogs, although further investigations to evaluate if proteinuria resolves following the resolution of these conditions are required to confirm any causal association.

## INTRODUCTION

Proteinuria, defined as the presence of increased quantities of protein in the urine, is classified as pathological or transient (physiological/functional), with pathological proteinuria suspected if persistent proteinuria is identified (Lees et al., 2005). Causes of pathological proteinuria are divided into pre‐renal, renal and post‐renal categories. Provided that pre‐renal and post‐renal causes are excluded, proteinuria can be considered renal in origin and further subclassified as renal glomerular or renal tubular proteinuria (Jepson, 2017). Measurement of urine protein:creatinine ratio (UPC) is the most commonly used method for quantifying proteinuria (Jepson, 2017), and in dogs, the International Renal Interest Society (IRIS) defines overt proteinuria in dogs as UPC >0.5 and borderline proteinuria as a UPC between 0.2 and 0.5. Studies in dogs, cats and humans have suggested that persistent proteinuria is associated with a greater frequency of renal morbidity, renal mortality and mortality of all causes (Jacob et al., 2005; Lees et al., 2005; Peterson et al., 1995; Syme et al., 2006). The pathophysiological mechanism underlying the association between proteinuria and progression of renal injury is postulated to involve excessive uptake of filtered proteins or protein‐bound substances by the renal tubules, resulting in damage to tubular structures and the development of interstitial fibrosis (Jacob et al., 2005; Vaden & Elliott, 2016; Walls, 2001).

Anecdotally, we have observed that proteinuria without known pathological pre‐renal, renal or post‐renal causes can be a finding in canine urine samples at our single referral institution, although renal pathology could not be fully excluded in these cases given that renal biopsy was not performed. If renal pathology was not present in these cases, then this could reflect physiological proteinuria, the mechanism for which is incompletely understood. It is suggested that physiological proteinuria may involve renal vasoconstriction, ischaemia, congestion, conformational changes in proteins associated with hyperlactatemia that increase permeability through the glomerular barrier, and increased permeability of the glomerular capillary membrane associated with increased sympathetic nervous system activity (Grauer, 2011; Poortmans et al., 2001; Suzuki & Ikawa, 1991). In people with systemic inflammatory disease, it has been proposed that the mechanism may be due to an overall increase in vascular permeability and thus an increase in glomerular permeability (Whittemore et al., 2006). Similarly, higher UPC, higher urine albumin: creatinine ratio and higher urinary retinol‐binding protein:creatinine ratios were observed in dogs with systemic inflammatory response syndrome when compared to healthy control dogs (Schaefer et al., 2011). The presence of mixed molecular weight proteins in the urine suggests that the proteinuria associated with systemic inflammation might be a combination of renal glomerular and tubular proteinuria, as high molecular weight (>80 kDa) proteins such as albumin are usually retained in the circulation by the glomerular barrier, and retinol binding protein, a low molecular weight (<60 kDa) protein, is usually reabsorbed in the proximal tubule. However, no studies have evaluated if there is an association between systemic inflammation (with or without pyrexia) and proteinuria in dogs, nor has the degree of proteinuria expected in these patients been reported in large numbers of patients.

The aim of the present study was to investigate if proteinuria (defined as UPC >0.2) observed in patients without known pathological pre‐renal, renal and post‐renal causes (but without definitive exclusion of renal pathology based on renal biopsy) was associated with serum C‐reactive protein concentrations (as a marker of systemic inflammation) and to document the severity of proteinuria in these cases. We also sought to evaluate which conditions are associated with proteinuria in the same group of dogs in order to identify other diseases that might be associated with proteinuria.

## MATERIALS AND METHODS

### Case selection and exclusion criteria

This was a retrospective, hypothesis‐generating study, in which dogs seen at a small animal referral hospital (Queen’s Veterinary School Hospital, Cambridge, UK) between January 2021 and December 2022 inclusive and that had available UPC and serum C‐reactive protein (CRP) data were included. All urine samples submitted to the laboratory at the referral hospital have urine protein:creatinine ratio measured as part of the routine urinalysis profile, and serum CRP concentrations are measured as part of the routine extended biochemistry profile in the laboratory. When multiple urinalyses per individual animal were performed, only results of the first urinalysis were included.

Cases with known pre‐renal, renal or post‐renal causes of proteinuria were excluded from the study, as summarised in Table 1. Dogs were excluded for suspected renal azotaemia based on a serum urea concentration above the laboratory reference interval (7.4 mmol/L) and/or serum creatinine concentration above the laboratory reference interval (136 μmol/L) with concurrent urine specific gravity <1.030. Dogs with renal or urinary system pathology noted on the clinical records (e.g. structural abnormalities of the renal or urinary tract based on imaging, urinalysis or biochemical abnormalities such as azotaemia) were excluded, although dogs with developmental abnormalities of the urinary system, for example, ectopic ureters, were included in the study provided they were not azotaemic or associated with an active sediment on urinalysis. Following review of medical records, dogs were excluded if they had known causes of proteinuria such as diabetes mellitus (Herring et al., 2014; Struble et al., 1998), hypercortisolism (Ortega et al., 1996), lymphoma (Di Bella et al., 2013), leukaemia, multiple myeloma, leishmaniasis (Koutinas & Koutinas, 2014), borreliosis (Dambach et al., 1997), shar‐pei fever (DiBartola et al., 1990) and systolic hypertension (defined as ≥160 mmHg) (Acierno et al., 2018). Dogs with hypercalcaemia were also excluded as hypercalcaemia is associated with nephrocalcinosis in cats (Tang et al., 2022). Dogs that had received corticosteroids within 7 days prior to urinalysis were also excluded (Waters et al., 1997).

**Table 1:** Summary of exclusion criteria for the study

Exclusion criteria	
Incomplete data	Absence of biochemistry or sCRP data
Active sediment	>5 RBC or WBC per 400× field
Bacteruria	Bacteria observed on urine sediment microscopy
Positive urine bacterial culture	
Positive haem dipstick panel	>trace result
Renal azotaemia	Serum creatinine concentration >153 μmol/L and/or serum urea concentration >10.7 mmol/L and USG <1.030
Renal or urinary system pathology	
Diabetes mellitus	
Hypercortisolism	
Lymphoma	
Leukaemia	
Multiple myeloma	
Leishmaniasis	
Shar‐pei fever	
Hypertension	Systolic blood pressure ≥160 mmHg
Hypercalcaemia	Serum total calcium concentration >2.7 mmol/L
Receiving corticosteroids	Within 7 days prior to urinalysis
Receiving tyrosine kinase inhibitors	Within 7 days prior to urinalysis

sCRP Serum C‐reactive protein, HPF High power field 40× objective, RBC Red blood cells, USG Urine specific gravity, WBC White blood cells

### Disease categorisation

For each individual case, the final diagnosis stated on the discharge paperwork was used to categorise the cases. Criteria for diagnosis of specific diseases of interest are summarised in Table S1. Dogs with more than one diagnosis stated in their medical records were categorised based on the diagnosis with the most relevance to the initial presenting clinical signs, as determined by the authors.

### Laboratory methods

Urine protein concentrations were measured using the pyrogallol red method and urine creatinine concentrations were measured using the Jaffe reaction on a Beckman AU480 analyser. A commercially available assay was used to measure serum CRP concentrations (sCRP, CRP assay, Randox), which was previously validated for use in dogs (Klenner et al., 2010). The limit of blank for sCRP in our laboratory was 2.2 mg/L and the upper limit of the laboratory reference interval was 8.2 mg/L (Klenner et al., 2010). For the purposes of statistical analyses, samples with sCRP <2.2 mg/L were assigned an arbitrary concentration of 1.1 mg/L and urine specific gravity (USG) >1.050 was assigned the arbitrary value of 1.051.

### Statistical analyses

The Kruskal–Wallis test with post hoc Mann–Whitney *U* test was used to compare clinicopathological variables between dogs with overt proteinuria (UPC >0.5), dogs with borderline proteinuria (UPC 0.21 to 0.5) and non‐proteinuric dogs (UPC ≤0.2). The Kruskal–Wallis test with post hoc Mann–Whitney *U* test was used to compare continuous variables between dogs that were non‐proteinuric (UPC ≤0.2) and dogs with borderline (UPC 0.212 to 0.5), mild (UPC 0.51 to 1.0), moderate (UPC 1.01 to 2) and marked (UPC >0.2) proteinuria. In order to evaluate the association between individual diseases and proteinuria (defined as UPC >0.2), the Fisher’s exact test was used to compare the prevalence of proteinuria in diseases for which there were ten or more cases included in the study (chronic enteropathy, pancreatitis, idiopathic epilepsy, intervertebral disc disease, mast cell tumour, chronic hepatitis, apocrine gland adenocarcinoma of the anal sac and portosystemic shunt) to the prevalence of proteinuria in the rest of the cohort. The correlation between UPC and CRP was determined using Spearman’s correlation coefficient. The association between different clinicopathological variables or specific diseases (with ten or more cases) and proteinuria was investigated using univariable and multivariable logistic regression analyses, with variables that showed a tendency towards a significant association (*P* < .2) with proteinuria on univariable analyses included in the multivariable logistic regression model. Data are presented as median [minimum to maximum], and statistical significance is defined as *P* < .05.

## RESULTS

A total of 1221 dogs had available UPC data in the study period. Of this cohort, 440 patients were defined as overtly proteinuric (UPC >0.5), 349 were borderline proteinuric and 432 were non‐proteinuric (UPC ≤0.2). The most common reasons for exclusion were evidence of post‐renal causes of proteinuria: urinary tract infection (UTI)/bacteriuria/positive urine culture (*n* = 85, 10%) or active sediment (*n* = 188, 22%) or the absence of biochemistry data within 24 hours of urinalysis (*n* = 186, 22%). A smaller proportion were excluded due to suspected renal azotaemia (*n* = 85, 10.1%), positive urine haem dipstick (> trace result) (*n* = 55, 6.5%) or lack of CRP data (*n* = 32, 3.8%). Reasons for exclusion for all dogs, stratified by proteinuric status, are summarised in Table 2.

**Table 2:** Table summarising the number of dogs meeting the exclusion criteria of the study in the overt proteinuric (UPC >0.5), borderline proteinuric (UPC 0.21 to 0.5) and non‐proteinuric cohorts (UPC ≤0.2)

Exclusion criteria	Overt proteinuric	Borderline proteinuric	Non‐proteinuric	Total
No serum biochemistry within 24 hours of urinalysis	45	93	48	186
No serum CRP concentration within 24 hours of urinalysis	6	10	16	32
Active sediment (>5 RBC/WBC per 400× field)	95	71	22	188
Positive urine bacterial culture/bacteriuria on urine sediment microscopy	49	24	12	85
Renal azotaemia (elevated serum urea and/or creatinine concentrations with USG <1.030)	52	16	17	85
Neoplasia (lymphoma/myeloma/leukaemia)	23	37	18	58
Hypercortisolism	11	4	4	19
Diabetes mellitus	11	2	10	23
Receiving corticosteroids	16	14	20	52
Receiving tyrosine kinase inhibitors	4	0	5	9
Systolic hypertension	7	1	3	11
Hypercalcaemia	3	3	2	8
Leishmaniasis	0	0	2	2
Shar‐pei fever	0	0	1	1
Urinary system pathology	11	4	10	25
Other/incomplete data set	1	1	0	2
Positive urine haem dipstick	35	12	8	55
Borreliosis	0	1	0	0
Totals	369	275	198	842

Dogs with incomplete data (with respect to C‐reactive protein [CRP] concentrations) and dogs with known causes of pre‐renal, renal or post‐renal proteinuria were excluded (although renal biopsy was not performed in any cases to fully exclude renal pathology)

CRP C‐reactive protein, USG Urine specific gravity, UTI Urinary tract infection

Following removal of animals according to the exclusion criteria, 379 dogs remained: 71 overtly proteinuric, 74 borderline proteinuric and 234 non‐proteinuric. Baseline clinicopathological data for the overtly proteinuric, borderline proteinuric and non‐proteinuric groups are summarised in Table 3. The overtly proteinuric group was significantly older than the borderline and non‐proteinuric groups (*P* = .003 and *P* < .001 respectively, Table 3) and had significantly lower serum creatinine concentrations than the borderline and non‐proteinuric groups (*P* < .001 for both comparisons, Table 3). The overtly proteinuric group also had lower USG compared to the non‐proteinuric group (*P* = .002, Table 3). The overtly proteinuric group had significantly higher urine protein concentrations compared to the borderline and non‐proteinuric groups (*P* < .001 for all comparisons, Table 3). Within the overtly proteinuric population, there were 37 dogs (52%) with mild proteinuria (UPC 0.51 to 1.0), 14 dogs (20%) with moderate proteinuria (UPC 1.01 to 2.0) and 20 dogs (28%) with severe proteinuria (UPC >2.0). Dogs with borderline proteinuria were younger than dogs with marked proteinuria (*P* = .015), but no differences in serum urea and creatinine concentrations were noted between the borderline, mild, moderate and marked proteinuria groups (Table 4). Serum CRP concentrations were significantly higher in dogs with mild proteinuria compared to dogs with borderline and marked proteinuria (*P* = .07 and *P* = .11 respectively, Table 4). Serum CRP concentrations were also significantly greater in dogs with borderline proteinuria compared to dogs classified as non‐proteinuric (*P* = .036, Table 4).

**Table 3:** Baseline clinicopathological data for dogs without known pre‐renal, renal or post‐renal causes of proteinuria that were overtly proteinuric (UPC >0.5), borderline proteinuric (UPC 0.21 to 0.5) and non‐proteinuric (defined as UPC ≤0.2) in the study cohort

	Overt proteinuric	Borderline proteinuric	Non‐proteinuric	Significance
Age (years)	9 [<1 to 15]^a,b^	7 [<1 to 15]^a^	6 [<1 to 16]^b^	*P* < .001
Urea (mmol/L)	4.7 [1.6 to 20.1]	4.6 [1.4 to 9.7]	5.1 [1.0 to 13.4]	*P* = .137
Creatinine (μmol/L)	75 [25 to 132]^a^	73 [35 to 116]^b^	85 [39 to 144]^a,b^	*P* < .001
USG	1.024 [1.002 to >1.050]^a^	1.025 [1.002 to >1.050]	1.033 [1.003 to >1.050]^a^	*P* = .004
Urine creatinine concentration (μmol/L)	8872 [588 to 38,994]^a^	12,176 [739 to 44,589]^b^	16,507 [790 to 51,482]^a,b^	*P* < .001
Urine protein concentration (g/L)	1.25 [0.04 to 10.09]^a,b^	0.40 [0.02 to 1.47]^b,c^	0.21 [<0.01 to 0.79]^a,c^	*P* < .001
UPC	0.98 [0.51 to 7.32]	0.28 [0.21 to 0.50]	0.11 [<0.01 to 0.2]	

UPC Urine protein:creatinine ratio, USG Urine specific gravity

Data are presented as median [minimum to maximum]. Comparisons between groups were made using the Kruskal–Wallis test. Groups with significant differences on post hoc Mann–Whitney *U* test (*P* < .05) are indicated by the same superscript letters

**Table 4:** Comparisons of age, serum urea, serum creatinine and serum CRP concentrations between proteinuric dogs with different severity of proteinuria

Parameter	Non‐proteinuric (*n* = 234)	Borderline proteinuria (*n* = 74)	Mild proteinuria (*n* = 37)	Moderate proteinuria (*n* = 14)	Marked proteinuria (*n* = 20)	Significance
Age (years)	6 [<1 to 16]^a,b,c^	7 [<1 to 15]^d^	9 [0 to 15]^a^	11 [1 to 13]^b^	9 [5 to 13]^c,d^	<.001
Serum urea concentrations (mmol/L)	5.1 [1.0 to 13.4]	4.6 [1.4 to 9.7]	4.1 [1.6 to 20.1]	5.2 [2.6 to 11.3]	4.8 [3.1 to 11.2]	.172
Serum creatinine concentrations (μmol/L)	85 [39 to 144]^a,b,c^	73 [35 to 116]^a^	72 [25 to 122]^b^	80 [44 to 101]	74 [54 to 132]^c^	<.001
Serum CRP concentrations (mg/L)	2.8 [<2.2 to 53.2]^a,b^	3.5 [<2.2 to 58.8]^a,c^	12.3 [<2.2 to 55.1]^b,c,d^	7.3 [<2.2 to 64.8]	<2.2 [<2.2 to 98.2]^d^	<.001

CRP C‐reactive protein

Non‐proteinuric dogs had urine protein:creatinine ratio (UPC) ≤0.2. Borderline proteinuria was defined as UPC of 0.21 to 0.5, mild proteinuria was defined as UPC of 0.5 to 1.0, moderate proteinuria was defined as UPC 1.01 to 2.0 and marked proteinuria was defined as UPC >2.0. Data are presented as median [minimum to maximum]. Comparisons were made between groups using the Kruskal–Wallis test with post hoc Mann–Whitney *U* test. Values with the same superscript letter indicate where significant differences were found between groups (*P* < .05)

Table S2 summarises the final diagnoses in the overtly proteinuric, borderline proteinuric, and non‐proteinuric dogs. Proteinuria (UPC >0.2) was less prevalent in dogs diagnosed with chronic enteropathy (11% [4/35] vs. 41% [141/344]; *P* < .001) compared to the rest of the population. Proteinuria was also more prevalent in dogs with chronic hepatitis (71% [10/14] vs. 37% [135/365]; *P* = .012) and tended to be more prevalent in dogs with pancreatitis (57% [12/21] vs. 37% [133/358]; *P* = .103) compared to the rest of the population. There was no difference in the prevalence of proteinuria between dogs with idiopathic epilepsy, intervertebral disc disease, mast cell tumours, apocrine gland adenocarcinoma of the anal sac or portosystemic shunts compared to the rest of the population (*P* > .2 for all comparisons).

Age, serum creatinine, globulin, CRP concentrations, serum alkaline phosphatase (ALP) activity and USG were all associated (*P* < .2) with proteinuria on univariable logistic regression analyses (Table 5) and hence included in the subsequent multivariable analyses. On multivariable logistic regression analysis, a diagnosis of chronic enteropathy remained independently associated with proteinuria even after adjustment for other confounding factors including age, serum creatinine, globulin and CRP concentrations, serum ALP activity and USG (OR = 0.21 [95% CI: 0.064 to 0.681]; *P* = .009, Table 6). Serum CRP concentration also remained independently associated with proteinuria after adjustment for other factors in the multivariable analysis (OR = 1.031 [95% CI: 1.012 to 1.051]; *P* = .001, Table 6). Diagnosis of chronic hepatitis was not independently associated with proteinuria after adjustment for other factors in the multivariable logistic regression analysis (OR = 3.04 [95% CI: 0.753 to 12.28]; *P* = .12). Similarly, a diagnosis of pancreatitis was not independently associated with proteinuria after adjustment for other factors in the multivariable logistic regression analysis (OR = 1.11 [95% CI: 0.38 to 3.25]; *P* = .85).

**Table 5:** Results of the univariable logistic regression analysis that evaluated the association between proteinuria (UPC >0.2) and age, serum concentrations of CRP, urea, creatinine, albumin and globulin, serum ALP activity and USG in dogs without known pre‐renal, renal and post‐renal causes of proteinuria (although renal pathology was not performed; therefore, renal pathology was not excluded entirely)

Variable	Beta	Odds ratio	95% CI for OR	Significance
Age (years)	0.092	1.096	1.037 to 1.158	*P* = .001
Serum CRP concentration (mg/L)	0.041	1.042	1.024 to 1.059	*P* < .001
Serum urea concentration (mmol/L)	<0.001	1.000	0.908 to 1.102	*P* = .996
Serum creatinine concentration (μmol/L)	−0.037	0.963	0.952 to 0.975	*P* < .001
Urine specific gravity (×1000)	−0.025	0.975	0.960 to 0.990	*P* = .001
Serum ALP activity (IU/L)	0.003	1.003	1.002 to 1.004	*P* < .001
Serum albumin concentration (g/L)	−0.024	0.976	0.938 to 1.016	*P* = .244
Serum globulin concentration (g/L)	0.074	1.077	1.035 to 1.122	*P* < .001

ALP Alkaline phosphatase, CRP C‐reactive protein, UPC Urine protein:creatinine ratio, USG Urine specific gravity

**Table 6:** Results of the multivariable logistic regression analysis that evaluated the association between proteinuria (UPC >.2) and diagnosis of chronic enteropathy, age, serum concentrations of CRP, creatinine and globulin, serum ALP activity and USG in dogs without known pre‐renal, renal or post‐renal causes of proteinuria (although renal biopsy was not performed; therefore, renal pathology was not excluded entirely)

	Beta	Sig	Odds ratio	95% CI for odds ratio	
				Lower	Upper
Age (years)	0.111	0.002	1.117	1.042	1.197
CRP (mg/L)	0.031	0.001	1.031	1.012	1.051
Creatinine (μmol/L)	−0.031	<0.001	0.970	0.956	0.983
Globulin (g/L)	0.015	0.528	1.015	0.969	1.064
ALP (IU/L)	0.001	0.003	1.001	1.000	1.002
Diagnosis of chronic enteropathy (referent to all other cases)	−1.565	0.009	0.21	0.064	0.681
USG (×1000)	−0.014	0.122	0.986	0.968	1.004
Constant	15.04	0.0115	3,415,514		

ALP Alkaline phosphatase, CRP C‐reactive protein, UPC Urine protein:creatinine ratio, USG Urine specific gravity

The Hosmer–Lemeshow test confirmed good model fit (P = .366)

**FIG 1:**
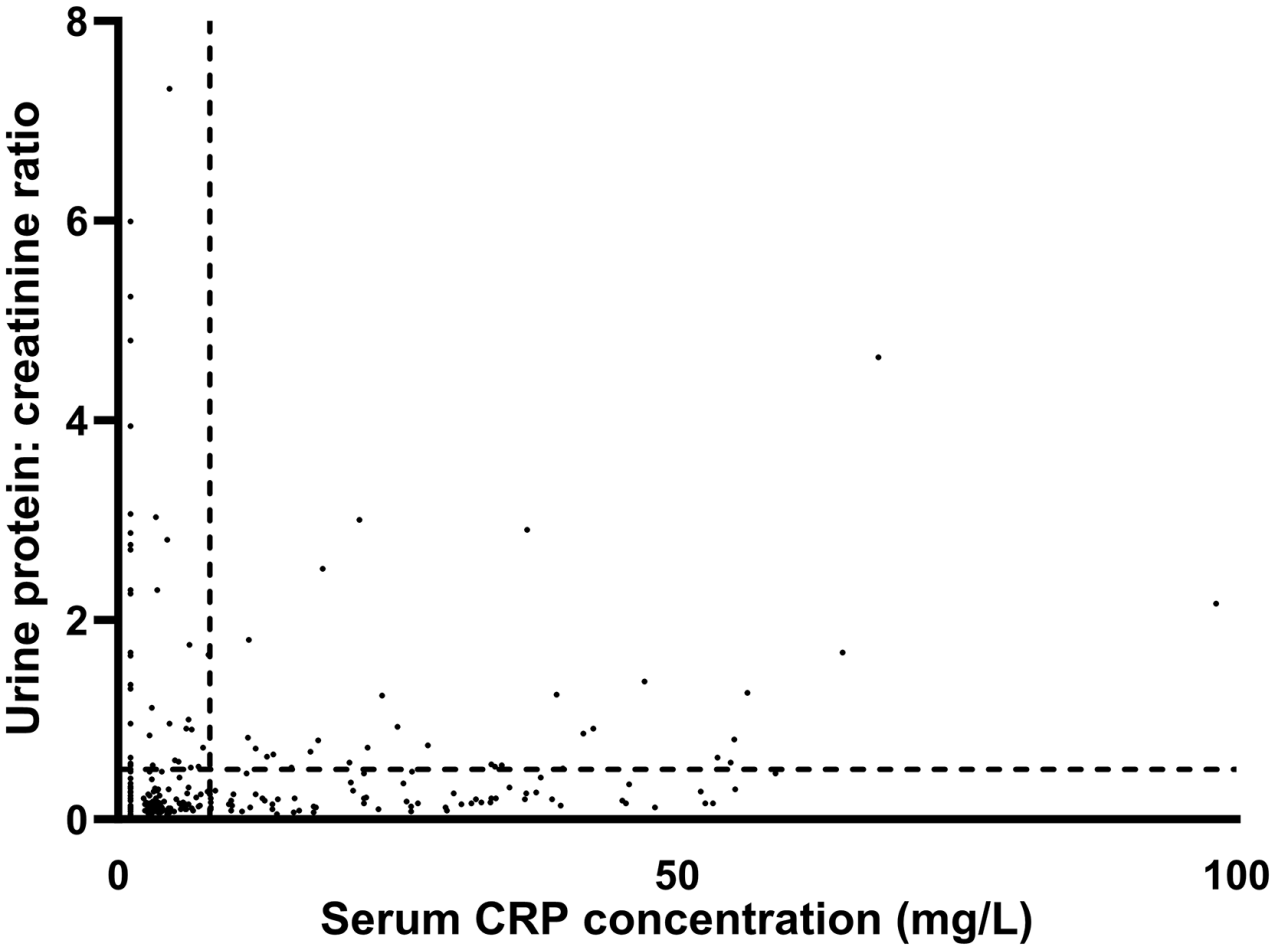
Scatter plot showing correlation between serum C‐reactive protein concentration (CRP) and urine protein:creatinine ratio (UPC). Serum CRP concentrations below the limit of blank of the assay (2.2 mg/L) were assigned the arbitrary value of 1.1 mg/L). Dotted line indicates the cut‐off for overt proteinuria as defined by the International Renal Interest Society (UPC >0.5) and the upper limit of the laboratory reference interval for CRP (8.2 mg/L). Correlations were assessed using Spearman’s correlation coefficient. Serum CRP concentrations were weakly positive correlated with UPC (*r*_*s*_ = 0.242; *P* < .001).

There was a weak positive correlation between serum CRP concentration and UPC (*r*_*s*_ = 0.242; *P* < .001, Fig 1). Borderline (UPC 0.21 to 0.5), mild (UPC 0.5 to 1.0), moderate (UPC 1.01 to 2.0) and marked (UPC > 2.0) proteinuria were observed in 25 (26%), 20 (21%), 6 (6%) and 5 (5%) dogs with serum CRP concentrations above the laboratory reference interval (>8.2 mg/L), respectively (*n* = 97). Borderline, mild, moderate and marked proteinuria were observed in 49 (17%), 17 (6%), 8 (3%) and 15 (5%) dogs with serum CRP concentrations within the laboratory reference interval (<8.2 mg/L), respectively (*n* = 282).

## DISCUSSION

The primary aims of the present hypothesis‐generating study were to examine if proteinuria (defined as UPC >0.2) was associated with serum CRP concentrations (as a marker of systemic inflammation) and to identify diseases that are associated with proteinuria in a group of dogs without known pathological pre‐renal, renal and post‐renal causes of proteinuria, although it should be noted that because renal biopsy was not performed in any patients, renal pathology could not be fully excluded. We found that there was a weak, positive association between serum CRP concentrations and UPC in dogs, and the presence of proteinuria was independently associated with serum CRP concentrations after adjustment for other clinicopathological variables that were associated with proteinuria in the univariable logistic regression analyses (with each 1 mg/L increase in serum CRP concentrations associated with a 3.1% increase in the odds of proteinuria). This could support the hypothesis that systemic inflammation might cause proteinuria in dogs and correlates with previous reports in humans and dogs that have documented an association between systemic inflammatory disease and proteinuria without a defined underlying mechanism (Reuben et al., 1982; Schaefer et al., 2011), although further longitudinal interventional studies would be required to establish a causal relationship between systemic inflammation and proteinuria. The mechanism underlying proteinuria in systemic inflammation remains poorly understood, although it has been suggested that a generalised increase in vascular permeability, perhaps associated with increased sympathetic tone, leads to an increase in glomerular permeability and subsequent leakage of proteins through the glomerular barrier (Grauer, 2011; Poortmans et al., 2001; Schaefer et al., 2011). Other proposed mechanisms include renal vasoconstriction, ischaemia, congestion and conformational changes in proteins that increase permeability through the glomerular barrier (Grauer, 2011; Suzuki & Ikawa, 1991). Further characterisation of the nature of proteinuria in dogs with systemic inflammation, for example, by urine protein electrophoresis and/or proteomic analysis of urine, and evaluation of renal histopathology (to evaluate structural changes in proteinuric dogs with systemic inflammation) would be warranted in order to establish the pathophysiology of proteinuria in these cases and fully exclude the presence of renal pathology; however, this was beyond the scope of this hypothesis‐generating retrospective study.

Dogs with albuminuria were at increased risk of death or euthanasia (odds ratio, 10.2; 95% confidence interval, 1.3 and 81.6) in one previous study (Vaden et al., 2010), suggesting that proteinuria may be a negative prognostic indicator. Further studies are warranted to establish if proteinuria associated with systemic inflammation is associated with prognosis in dogs and to examine if proteinuria resolves following resolution of systemic inflammation in these cases.

Proteinuria was less prevalent in cases diagnosed with chronic enteropathy in our study, which conflicts with previous data suggesting that overt proteinuria was present in approximately 40% of non‐azotaemic dogs with chronic enteropathy (Gori et al., 2022). In our population, proteinuria was only observed in 11% of dogs with chronic enteropathy, and a diagnosis of chronic enteropathy remained at significantly reduced odds of proteinuria after adjustment for other factors in the multivariable analysis. Our findings could suggest that a diagnosis of proteinuria (UPC >0.2) in dogs with chronic enteropathy might warrant further investigations as to the cause of proteinuria in these cases.

There were various limitations to our study, many of which are inherent given the retrospective nature of the study. Firstly, its retrospective nature meant that some dogs were excluded due to incomplete data, which could have biased our findings, although the number of cases with incomplete data that were excluded was relatively low (two cases). Urine sampling was not performed in all dogs admitted to our hospital during the study period, but rather was performed in animals for which urinalysis was considered useful in the diagnostic investigations; however, it should be noted that UPC analysis was routinely performed on all urine samples submitted for analysis. Bacterial culture was not performed on all urine samples to fully exclude the presence of bacteriuria, which could also contribute to post‐renal proteinuria (Broadbridge & Williams, 2023). Urinary tract imaging (to exclude renal or urinary tract pathology) was not performed in all cases; therefore, the presence of urinary tract disease could not be fully ruled out in all cases. In addition, IRIS stage 1 CKD and primary glomerulopathies have not been excluded in any cases, as histopathological evaluation of the kidneys was not performed in any patients. Further characterisation of proteinuria by urine protein electrophoresis was not possible due to the retrospective nature of the study, but would have been helpful to establish the type of proteinuria present (i.e. high molecular weight proteinuria vs. low molecular weight proteinuria) and give insights into the pathophysiology of the proteinuria that was observed. Hypertension (a potential cause of proteinuria) was also not ruled out in all cases, since blood pressure measurements were only performed at the discretion of the attending clinicians. The prevalence of hypertension in the general canine population is expected to be low (Bodey & Michell, 1996), and so may not be a significant confounding factor; although the prevalence of hypertension in a hospitalised patient population may be higher than in the general canine population. In addition, exclusion of all diseases that might cause proteinuria (e.g. hypercortisolism) was not possible given the retrospective nature of the study; therefore, the presence of diseases such as hypercortisolism could not be excluded. We also only excluded patients for which steroids had been discontinued at least 7 days prior to UPC analysis, and it is possible that UPC may have been increased in dogs that had been treated with steroids between 7 and 14 days prior to UPC analysis, given that currently there is only evidence to support resolution of proteinuria 14 days after cessation of steroid therapy (Mantelli et al., 2022). Persistence of proteinuria, defined as detection of proteinuria on ≥3 occasions, ≥2 weeks apart (Grauer, 2011), was also not demonstrated as part of our study, with UPC only noted on a single urine sample; further studies to demonstrate if persistent proteinuria is observed in these patients would be warranted. In addition, daily variability in UPC might lead to a change in the classification of proteinuria if additional samples were evaluated. Dogs with diseases that can be associated with pre‐renal proteinuria (immune‐mediated haemolytic anaemia (IMHA) and precursor‐targeted immune‐mediated anaemia (PIMA)) were included in the study provided that they did not have evidence of haemoglobinuria on urinalysis (based on results of dipstick analysis). There is the possibility that some of these cases may have had low‐level haemoglobinuria, below the limit of detection of the dipstick, which might artefactually increase UPC; however, while blood contamination has been shown to increase UPC, only macroscopic haematuria is anticipated to directly produce significant proteinuria (UPC >.5) (Jillings et al., 2019), and so we do not consider this to be a significant confounding factor. A final limitation of our study is that diagnosis of pancreatitis was made routinely following suggestive clinical signs, clinicopathological findings and concordant imaging findings rather than by histopathology; therefore, a definitive diagnosis nor characterisation of chronic pancreatitis could be made in all cases.

In conclusion, the results of this hypothesis‐generating retrospective study suggest that proteinuria might be associated with systemic inflammation in dogs without known pre‐renal, renal or post‐renal causes of proteinuria, although it should be noted that in light of the lack of renal biopsy in any patients, the presence of renal pathology was not definitively excluded. Further studies are needed to develop a better understanding of the pathophysiology of proteinuria in systemic inflammation and to evaluate if this proteinuria resolves following the resolution of systemic inflammation (and thus support a causal association between the proteinuria and inflammation).

### Author contributions

**E. Ruane:** Conceptualization (supporting); data curation (lead); writing – original draft (lead); writing – review and editing (supporting). **M. M. A. Rodgers:** Data curation (equal); investigation (equal). **C. H. Z. Hare:** Conceptualization (supporting); methodology (supporting); writing – original draft (supporting); writing – review and editing (supporting). **K. E. McCallum:** Conceptualization (supporting); methodology (supporting); writing – original draft (supporting); writing – review and editing (supporting). **T. L. Williams:** Conceptualization (lead); data curation (supporting); formal analysis (lead); investigation (supporting); supervision (lead); writing – original draft (supporting); writing – review and editing (lead).

### Conflict of interest

E. Ruane – No conflicts of interest have been declared. M. M. A. Rodgers – No conflicts of interest have been declared. C. H. Z. Hare – Speaker fees – improve International. K. E. McCallum – Speaker fees – BSAVA PetSavers, VVC, SEVC, Protexin, TSAVA, University of Sydney; grant funding – ECVCP. T. L. Williams – Consultancy – Purina, Petmedix Ltd, Carus Animal Health; speaker fees – BSAVA PetSavers, ACVIM, ECVIM, ESVE, ESVCP, Dechra Pharmaceuticals, MV Minds; grant funding – BSAVA PetSavers, Academy of Medical Sciences, PetPlan Charitable Trust, Pets at Home, The Urology Foundation; other financial or in‐kind support – IDEXX Laboratories, University of Cambridge.

## Funding

This study did not receive any support in the form of grants or equipment.

## Supporting information


**Table S1.** Diagnostic criteria for diagnosis of diseases with more than 10 cases.

**Table S2.** Final diagnoses of dogs without known pre‐renal, renal and post‐renal causes of proteinuria (although it should be noted that renal biopsy was not performed in any cases therefore renal pathology was not fully excluded). Dogs were stratified into overt proteinuria (defined as urine protein:creatinine ratio [UPC] >0.5), borderline proteinuria (UPC 0.21 to 0.5) and non‐proteinuric groups (UPC ≤0.2) within each disease category. Specific disease diagnostic criteria for diseases shown in bold (those with >10 cases) are summarised in Table S1.

## Data Availability

We will make pertinent data available after publication of the manuscript upon request.
