# Supplementary material for: Investigation of association between serum C‐reactive protein concentrations and proteinuria in dogs
Source: J Small Anim Pract. 2025 Oct 29;67(3):235–42. doi: 10.1111/jsap.70040 (PMC12968500; doi:10.1111/jsap.70040)
Supplement: Supplementary file 1 — Table S1. Diagnostic criteria for diagnosis of diseases with more than 10 cases. [file JSAP-67-235-s002.docx]

**Diagnostic criteria for diagnosis of diseases with more than 10 cases**

- Apocrine gland adenocarcinoma of the anal sac – cytological diagnosis
- Mast cell tumour – cytological diagnosis
- Chronic enteropathy - Gastrointestinal signs >3 weeks duration with no imaging or biochemical findings consistent with extra-intestinal disease
- Chronic hepatitis – diagnosed on histopathology
- Intervertebral disc disease – confirmed by MRI

Basis of diagnosis of pancreatitis is shown on table below. Pancreatitis was diagnosed based on compatible findings on abdominal ultrasound along with either cranial abdominal pain or elevated serum lipase activity:

| **Presenting Complaint** | **Cranial abdominal pain** | **Serum lipase activity (DGGR assay)** | **Pancreatic ultrasound findings** |
| --- | --- | --- | --- |
| Vomiting, lethargy, inappetence, PUPD | Yes | Increased | Enlarged |
| Lethargy, anorexia,  PUPD | Yes | Increased | Hypoechoic with hyperechoic peripancreatic fat |
| Vomiting, abdominal pain, tachycardia, anorexia | Yes | Increased | Enlarged, hyperechoic |
| Hyporexia, PUPD | No | Increased | Markedly hyperechoic right limb |
| Vomiting, diarrhoea,  weight loss, abdominal pain | Yes | Increased | Hyperechoic patches |
| Vomiting, abdominal pain, hyporexia | Yes | Increased | Hyperechoic (with hypoechoic regions), irregular margins and surrounding hyperechoic mesentery |
| Vomiting | Yes | Increased | Speckled with heterogenous echogenicity |
| Vomiting, diarrhoea | No | Increased | Enlarged, diffusely heterogeneously speckled |
| Vomiting, diarrhoea | Yes | Within RI | Hyperechoic |
| Vomiting and lethargy | Yes | Increased | Enlarged, hypoechoic, irregular margins and hyperechoic mesenteric fat surrounding |
| Diarrhoea, lethargy | Yes | Within RI | Hyperechoic left limb & speckled right limb |
| Vomiting, lethargy, hyporexia | Yes | Within RI | Hypoechoic with hyperechoic fat |
| Vomiting, diarrhoea, weight loss, hyporexia, lethargy | No | Increased | Patchy hyperechoic right limb |
| Vomiting, diarrhoea, hyporexia, lethargy | No | Increased | Hypoechoic |
| Vomiting, diarrhoea, hyporexia | No | Increased | Hypoechoic with hyperechoic adjacent mesentery |
| Vomiting | Yes | Within RI | Hyperechoic, heterogenous |
| Vomiting and diarrhoea | No | Within RI | Hyperechoic left limb, surround by hyperechoic lobulated fat. Small amount of anechoic free fluid. |
| Vomiting, diarrhoea, abdominal pain | No | Increased | Hyperechoic left limb |
| Vomiting, lethargy, hyporexia | No | Increased | Left limb hypoechoic with hyperechoic surrounding fat |
| Chronically elevated cPLI (without clinical signs) | No | Increased | Multifocal hypoechoic specks in parenchyma |
| Vomiting, diarrhoea, anorexia | No | Increased | Hypoechoic parenchyma with hyperechoic mesentery |

Basis of diagnosis of idiopathic epilepsy cases is shown on table below:

| **Age at seizure onset** | **Unmet Tier 1 criteria** | **Tier 2 criteria** | | | **Confidence interval** |
| --- | --- | --- | --- | --- | --- |
|  |  | **Normal MRI** | **Normal CSF** | **Normal bile acid stimulation test** |  |
| 6 years | None | Yes | Yes | Yes | Tier 2 |
| 3 years | None | Yes | Yes | Yes | Tier 2 |
| 2 years | None | Not performed | Not performed | Yes | Tier 1 |
| 3 years | None | Yes | Yes | Not performed | Tier 2 |
| 3 years | Fasted BA/NH3 | Yes | Yes | Not performed | Tier 1 |
| 1 year | None | Yes | Yes | Not performed | Tier 2 |
| 3 years | None | Yes | Yes | Not performed | Tier 2 |
| 4 years | None | Yes | Yes | Not performed | Tier 2 |
| 2 years | None | Yes | Yes | Not performed | Tier 2 |
| 1 year | None | Yes | Yes | Yes | Tier 2 |
| 6 years | None | Yes | Not performed | Yes | Tier 2 |
| 7 years | >6 years at onset | Yes | Not performed | Yes | Tier 1 |
| 10 years | >6 years at onset | Not performed | Not performed | Yes | Tier 1 |
| 6 years | None | Not performed | Not performed | Yes | Tier 1 |
| 4 years | None | Yes | Not performed | Yes | Tier 2 |
| 4 years | None | Yes | Yes | Yes | Tier 2 |
| 3 years | None | Yes | Yes | Not performed | Tier 2 |
| 1 year | None | Yes | Yes | Yes | Tier 2 |
| 5 years | None | Yes | Yes | Yes | Tier 2 |
| 3 years | None | Yes | Not performed | Yes | Tier 2 |
| 2 years | None | Yes | Yes | Yes | Tier 2 |

Tier 1 confidence interval: 6 months to 6 years old at onset, unremarkable inter-ictal clinical exam, minimum of 2 seizures >24hrs apart, haematology and biochemistry (including fasting bile acid /ammonia concentration) and urinalysis.

Tier 2 confidence interval: All Tier 1 criteria + MRI **and** CSF/BAST.

Basis for diagnosis of portosystemic shunt is shown on the table below:

| **Clinical**  **signs** | **Increased ammonia** | **Bile**  **acids** | **Other biochemical abnormalities** | **Imaging**  **modality** | **Imaging findings** | **Shunt type** |
| --- | --- | --- | --- | --- | --- | --- |
| Lethargy, hyporexia, abdominal pain | No | Increased pre- and post-prandially | -Increased ALP activity  -Hypoalbuminaemia | Ultrasound | Extrahepatic shunt | Congenital |
| Vomiting, stunted growth, circling, head pressing, PUPD | Yes | Increased pre- and post-prandially | -High ALT & ALP  activities  -Hypoalbuminaemia  -Decreased urea  concentration | Ultrasound | Intrahepatic shunt | Congenital |
| Vomting, vacancy, dullness | No | Not tested | -Increased ALT & ALP  activities  -Hypoglycaemia | Ultrasound | Extrahepatic shunt and microhepatica | Congenital |
| Lethargy, head pressing, PUPD, abdominal pain | No | Increased pre- and post-prandially | Increased ALT & ALP  activities | Ultrasound | Extrahepatic shunts and microhepatica | Acquired |
| Vomiting, diarrhoea, lethargy, failure to thrive | No | Marked pre-prandial increase | -Increased ALT & ALP  activities  -Hypoalbuminaemia | Ultrasound | Extrahepatic shunt | Congenital |
| Circling, vacancy, inappetence | Yes | Not tested | -Increased ALT & ALP  activities  -Hypoalbuminaemia  -Decreased urea concentration  -Hypocholesterolaemia | Ultrasound | Extrahepatic shunt | Acquired |
| Obtundation, pica, disorientation | Yes | Increased pre- and post-prandially | Hypoalbuminaemia | Ultrasound | Extrahepatic shunts | Acquired |
| Drooling, pacing, circling | No | Increased pre-prandially | -Decreased urea concentration  -Increased ALT activity | Ultrasound | Extrahepatic shunt | Congenital |
| Vomiting, seizures, diarrhoea | Yes | Increased pre- and post-prandially | None | Ultrasound | Extrahepatic shunt | Congenital |
| Vomiting, diarrhoea, PUPD, weight loss | Yes | Not tested | -Hypoalbuminaemia  -Decreased urea  concentration  -Increased ALT activity | Computed tomography | Extrahepatic shunt | Congenital |
| Lethargy, inappetence, seizures, PUPD | Yes | Increased pre-prandially | -Hypoalbuminaemia  -Increased ALT and  ALP activities -Hypocholesterolaemia | Ultrasound | Extrahepatic shunt | Congenital |
| Stunted growth, tachypnoea | No | Increased pre- and post-prandially | -Increased ALP activity  -Hypoalbuminaemia | Ultrasound | Extrahepatic shunt | Congenital |
