# Supplementary material for: Investigation of association between serum C‐reactive protein concentrations and proteinuria in dogs
Source: J Small Anim Pract. 2025 Oct 29;67(3):235–42. doi: 10.1111/jsap.70040 (PMC12968500; doi:10.1111/jsap.70040)
Supplement: Supplementary file 2 — Table S2. Final diagnoses of dogs without known pre‐renal, renal and post‐renal causes of proteinuria (although it should be noted that renal biopsy was not performed in any cases therefore renal pathology was not fully excluded). Dogs were stratified into overt proteinuria (defined as urine protein:creatinine ratio [UPC] >0.5), borderline proteinuria (UPC 0.21 to 0.5) and non‐proteinuric groups (UPC ≤0.2) within each disease category. Specific disease diagnostic criteria for diseases shown in bold (those with >10 cases) are summarised in Table S1. [file JSAP-67-235-s001.docx]

Supplementary Table 2. Final diagnoses of dogs without known pre-renal, renal and post-renal causes of proteinuria (although it should be noted that renal biopsy was not performed in any cases therefore renal pathology was not fully excluded). Dogs were stratified into overt proteinuria (defined as urine protein:creatinine ratio [UPC] >0.5), borderline proteinuria (UPC 0.21-0.5) and non-proteinuric groups (UPC ≤0.2) within each disease category. Specific disease diagnostic criteria for diseases shown in bold (those with >10 cases) are summarised in Supplementary Table 1.

| **Disease Classification** | **Total case number** | **UPC >0.5** | **UPC 0.21-0.5** | **UPC ≤0.2** |
| --- | --- | --- | --- | --- |
| **Chronic enteropathy** | **35** | **0** | **4** | **31** |
| **Pancreatitis** | **21** | **11** | **1** | **9** |
| **Epilepsy** | **21** | **0** | **5** | **16** |
| **Mast cell tumour** | **17** | **3** | **1** | **13** |
| **Intervertebral disc disease** | **14** | **1** | **4** | **9** |
| **Chronic hepatitis** | **14** | **5** | **5** | **4** |
| **Apocrine gland adenocarcinoma of the anal sac** | **14** | **2** | **4** | **8** |
| **Portosystemic shunt** | **12** | **2** | **4** | **6** |
| Investigations into abnormal episodes or collapse (no cause identified) | 12 | 2 | 2 | 8 |
| Chronic hepatopathy | 8 | 0 | 7 | 1 |
| Dietary intolerance/indiscretion/gastritis | 8 | 1 | 2 | 5 |
| Possible pancreatitis (did not meet all criteria) | 8 | 5 | 0 | 3 |
| Carcinoma | 6 | 4 | 2 | 0 |
| Steroid-responsive meningitis-arteritis | 5 | 0 | 2 | 3 |
| Pneumonia | 4 | 1 | 1 | 2 |
| Gall bladder mucocele | 2 | 1 | 1 | 0 |
| Biliary sludge | 1 | 0 | 0 | 1 |
| Meningoencephalitis of unknown origin | 4 | 2 | 1 | 1 |
| Immune-mediated polyarthritis | 4 | 1 | 2 | 1 |
| Ectopic ureters | 3 | 2 | 0 | 1 |
| Seizures not classified/confirmed as idiopathic epilepsy | 3 | 0 | 1 | 2 |
| Investigation of gastrointestinal signs | 3 | 0 | 1 | 2 |
| Acute hepatitis/hepatopathy | 3 | 1 | 1 | 1 |
| Adrenal mass (no diagnosis) | 3 | 0 | 0 | 3 |
| Brain tumour (no diagnosis) | 4 | 0 | 2 | 2 |
| Suspected IVDD (no MRI) | 2 | 0 | 1 | 1 |
| Lumbosacral stenosis | 3 | 0 | 0 | 3 |
| Myxomatous mitral valve disease | 2 | 0 | 1 | 1 |
| Precursor-targeted immune-mediated anaemia | 2 | 0 | 1 | 1 |
| Diabetes insipidus | 3 | 1 | 1 | 1 |
| Hip dysplasia | 3 | 0 | 0 | 3 |
| Gastric/intestinal ulceration | 2 | 0 | 0 | 2 |
| Foreign body | 4 | 0 | 1 | 3 |
| Anaemia (no cause identified) | 3 | 0 | 1 | 2 |
| Hepatic neoplasm (no diagnosis) | 3 | 2 | 0 | 1 |
| Histiocytic sarcoma | 2 | 1 | 1 | 0 |
| AV block | 1 | 0 | 0 | 1 |
| Pulmonary mass | 2 | 1 | 0 | 1 |
| Squamous cell carcinoma | 2 | 1 | 1 | 0 |
| Cognitive dysfunction | 1 | 0 | 0 | 1 |
| Fibrocartilagenous embolism | 2 | 0 | 1 | 1 |
| Idiopathic head tremors | 2 | 0 | 0 | 2 |
| Vestibular disease | 2 | 0 | 1 | 1 |
| Insulinoma | 2 | 1 | 0 | 1 |
| Intrapelvic bladder | 2 | 0 | 0 | 2 |
| USMI | 3 | 0 | 1 | 2 |
| Osteomyelitis | 2 | 1 | 0 | 1 |
| Megaoesophagus | 2 | 0 | 0 | 2 |
| Oesophagitis | 2 | 0 | 0 | 2 |
| Failure to thrive | 2 | 0 | 1 | 1 |
| Pyrexia/inflammation of unknown origin | 3 | 2 | 1 | 0 |
| Anaplastic sarcoma | 1 | 1 | 0 | 0 |
| Intestinal mass (no diagnosis) | 1 | 0 | 0 | 1 |
| Jejunal mass (no diagnosis) | 1 | 0 | 0 | 1 |
| Melanoma | 1 | 0 | 0 | 1 |
| Osteosarcoma | 1 | 1 | 0 | 0 |
| Splenic sarcoma | 1 | 0 | 0 | 1 |
| Spinal neoplasia | 1 | 0 | 0 | 1 |
| Thyroid tumour | 1 | 0 | 0 | 1 |
| Trigeminal nerve neoplasm | 1 | 0 | 0 | 1 |
| Seborrhoeic dermatitis | 1 | 0 | 0 | 1 |
| Meningioma | 1 | 0 | 0 | 1 |
| Digital keratoma | 1 | 0 | 0 | 1 |
| Surgical site infection | 1 | 0 | 0 | 1 |
| Brain cyst | 1 | 0 | 1 | 0 |
| Discospondylitis | 1 | 0 | 0 | 1 |
| Paroxysmal dyskinesia | 1 | 0 | 0 | 1 |
| Oral foreign body | 1 | 0 | 0 | 1 |
| Spinal anomaly | 1 | 0 | 0 | 1 |
| Steroid-responsive tremor syndrome | 1 | 0 | 0 | 1 |
| Portal hypertension | 1 | 0 | 0 | 1 |
| Aortic valve stenosis | 1 | 1 | 0 | 0 |
| Brachycephalic obstructive airway syndrome | 1 | 1 | 0 | 0 |
| Infectious tracheobronchitis | 1 | 0 | 0 | 1 |
| Interstitial pulmonary disease | 1 | 1 | 0 | 0 |
| Myocarditis | 1 | 0 | 0 | 1 |
| Thrombus in caudal vena cava | 1 | 1 | 0 | 0 |
| Tracheal collapse | 1 | 0 | 0 | 1 |
| Discoid lupus erythematosus | 1 | 0 | 0 | 1 |
| Immune-mediated haemolytic anaemia | 1 | 0 | 0 | 1 |
| Immune-mediated thrombocytopenia | 1 | 0 | 0 | 1 |
| Lymphadenitis | 1 | 0 | 1 | 0 |
| Pemphigus foliaceous | 1 | 0 | 0 | 1 |
| Peri-anal furunculosis | 1 | 1 | 0 | 0 |
| Atypical hypoadrenocorticism | 1 | 0 | 1 | 0 |
| Balanoposthitis (cystocentesis sample) | 1 | 0 | 0 | 1 |
| Pollakiuria (no cause identified) | 1 | 0 | 1 | 0 |
| Urinary incontinence | 1 | 1 | 0 | 0 |
| Lameness (no cause identified) | 1 | 0 | 0 | 1 |
| Osteoarthritis | 2 | 0 | 0 | 2 |
| Adverse reaction to NSAIDs | 1 | 0 | 0 | 1 |
| Renal monitoring in asymptomatic dog | 1 | 0 | 0 | 1 |
| Weight gain due to overfeeding | 1 | 0 | 0 | 1 |
| Splenic mass (no histopathology/cytology) | 1 | 0 | 1 | 0 |
| Pre-op cryptorchid castrate | 1 | 0 | 0 | 1 |
| Right tibial fracture repair (post-op check) | 1 | 1 | 0 | 0 |
| Septic retroperitonitis (no cause identified) | 1 | 0 | 1 | 0 |
| Sublumbar abscess (secondary to foreign body) | 1 | 0 | 0 | 1 |
| Rodenticide toxicity | 1 | 0 | 0 | 1 |
| Hypereosinophilic syndrome | 1 | 0 | 0 | 1 |
| Tetraparesis | 1 | 0 | 0 | 1 |
| Vasculitis (ischaemic dermatopathy) | 1 | 0 | 0 | 1 |
| Polyradiculoneuritis | 1 | 1 | 0 | 0 |
| Vertebral body neoplasia | 1 | 0 | 0 | 1 |
| Subarachnoid diverticulum | 1 | 0 | 1 | 0 |
| Mediastinitis (secondary to FB) | 1 | 0 | 0 | 1 |
| Peripheral nerve sheath tumour | 1 | 0 | 0 | 1 |
| Metastatic neuroendocrine neoplasia | 2 | 0 | 0 | 2 |
| No final diagnosis reached | 32 | 7 | 3 | 22 |
